# Partial Mandibulectomy Rehabilitation of Keratocystic Odontogenic Tumour Case in Neutral Zone

**DOI:** 10.1155/2018/8918673

**Published:** 2018-06-21

**Authors:** Mohamad Syahrizal Halim, Tengku Fazrina Tengku Mohd Ariff

**Affiliations:** ^1^Conservative Dentistry Unit, School of Dental Sciences, Universiti Sains Malaysia, Health Campus, 16150 Kota Bharu, Kelantan, Malaysia; ^2^Centre for Restorative Dentistry Studies, Faculty of Dentistry, Jalan Hospital, Universiti Teknologi MARA, Sungai Buloh Campus, 47000 Sungai Buloh, Selangor Darul Ehsan, Malaysia

## Abstract

Restoring the patient's missing dentition secondary to partial mandibulectomy of KCOT is important to improve function and aesthetics. The patient presented with a significant loss of alveolar bone which makes the fabrication of rehabilitation prosthesis a significant challenge. A neutral-zone impression technique is helpful in determining the exact space to be restored without compromising aesthetics and it avoids functional muscle displacement that may displace the prosthesis. This article describes the neutral zone impression technique to record a patient's functional muscular movement in guiding the setting of acrylic teeth and denture flange in the neutral zone area. This technique is very useful for postsurgical cases with significant loss of alveolar bone.

## 1. Introduction

Keratocystic odontogenic tumour (KCOT) is a benign intraosseous neoplasm which can occur in a unicystic or multicystic form and originate from odontogenic tissue [1]. Originally known as an odontogenic keratocyst, WHO has reclassified this lesion into a new name to reflect the aggressive nature of this benign neoplasm. Its aggressive and infiltrative behaviour is due to its propensity to grow inside the jaws, with minimal expansion [2]. These features warrant aggressive surgical removal to prevent recurrence of the lesion.

KCOT surgical treatment may cause the patient to have functional and aesthetic problems. This is due to the aggressive surgical treatment performed by the surgeon which includes at least 2 mm surgical removal from the margin's lesion. This is done to prevent recurrence as the lesion can infiltrate the head and neck bone. The overall rate of occurrences of postsurgical treatment of KCOT are 23.09% [3].

Rehabilitation of patient postsurgical segmental mandibulectomy gives significant challenges to a dentist. The patient may present with a significant loss in vertical and horizontal alveolar bone which will make the fabrication of rehabilitation prosthesis a setback. After a surgical procedure, the patient is left with a space that used to be occupied with teeth and alveolus. This loss in space will allow the tongue and buccal mucosa tissue to occupy the space which will make the neutral zone smaller. A problem may also arise since there is no alveolar ridge to serve as a guide in the placement of the prosthetic teeth during acrylic denture setting. Minute displacement in the functional space will compromise denture stability, disturb patient speech, and interfere with patient masticatory function.

Preferably, the loss in vertical and horizontal bone should be replaced with vascularized bone graft or alloplastic material, which may involve another surgery for the former graft. Most of the patients who have been diagnosed with this neoplasm are reluctant to undergo through the surgical and emotional stress from another surgery. So this method of rehabilitation by removable of a partial denture recorded in the neutral zone is an alternative for patients who refuse such emotional and physical stress. This method will also serve as an interim prosthesis before future implant treatment consideration.

The purpose of this paper is to highlight the importance of the neutral zone for a mandibular partial denture in the rehabilitation of partial mandibulectomy of KCOT. This ensures that the polished surface of the denture does not encroach the functional movement on the lingual and buccal musculature and eventually minimizes denture displacement.

### 1.1. Case

**Figure 1:**
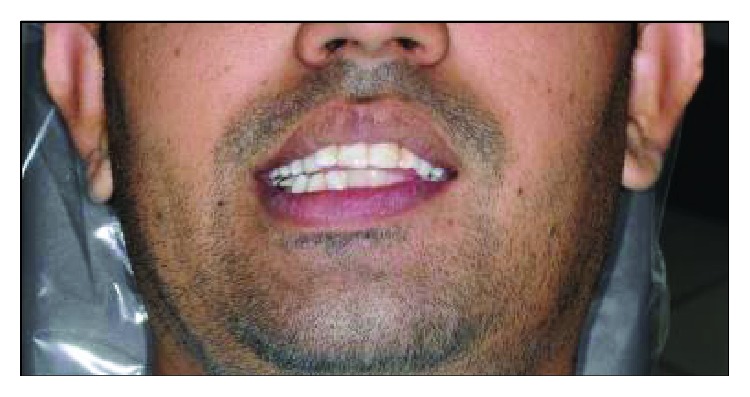
Facial profile at initial presentation.

A 29-year-old Pakistani male was referred to the Oral Surgery Department for rehabilitation of the left edentulous mandible secondary to partial mandibulectomy surgery. He had undergone two operations for the left body of a mandible keratocyst odontogenic tumour (KCOT). The patient has been diagnosed with (KCOT) in Pakistan, where he received his first surgical treatment. A second surgical partial mandibulectomy was attempted due to recurrence. He has been reviewed regularly and he requested to have a replacement of his missing teeth on the lower left side due to a difficulty in eating and the effect on his appearance (Figure 1).

**Figure 2:**
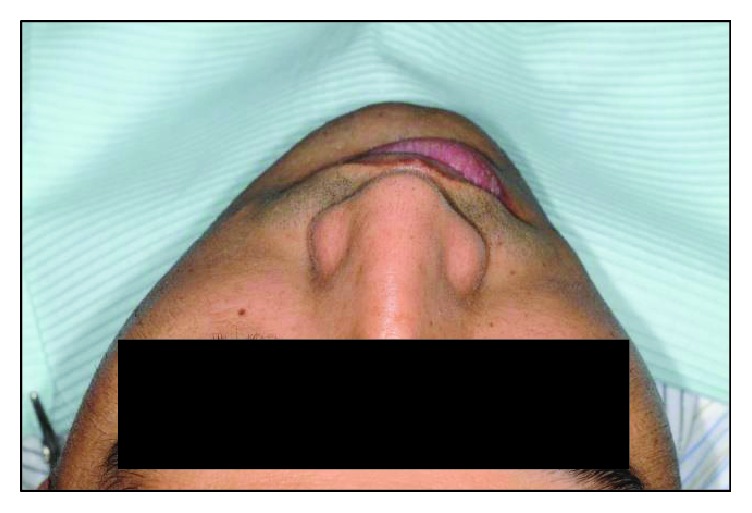
Coronal view profile at initial presentation.

Clinical examination reveals an asymmetrical face with a slightly depressed left lower body of the mandible on a class I skeletal pattern (Figures 1 and 2). The patient reported an absence of paraesthesia on the left mandible. The smile line was high exposing the gingiva on the upper maxillary incisors.

**Figure 3:**
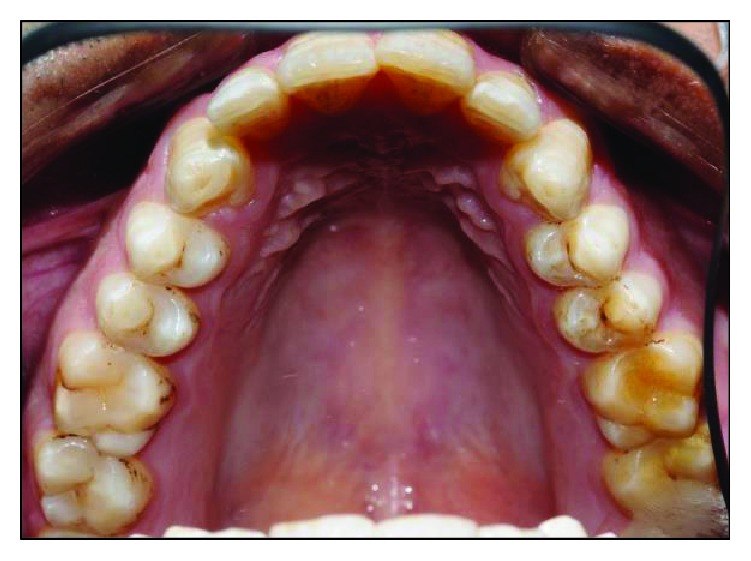
Preoperative maxillary occlusal view.

**Figure 4:**
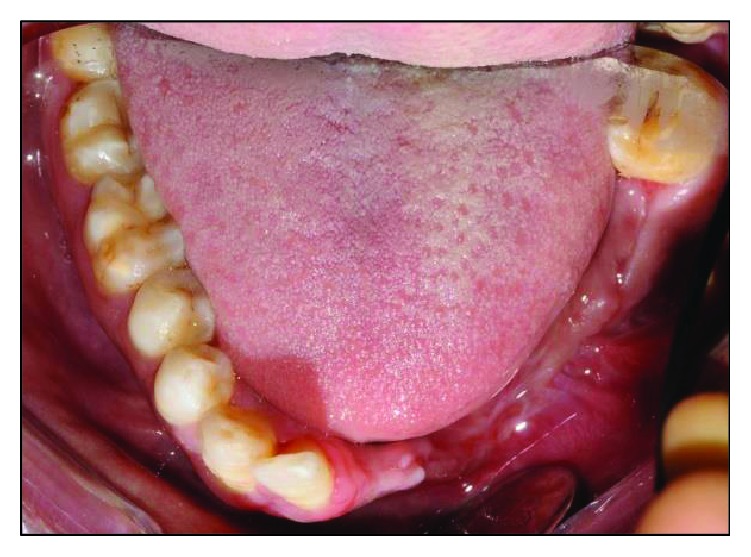
Preoperative mandibular occlusal view, note the tongue occupying the edentulous area.

**Figure 5:**
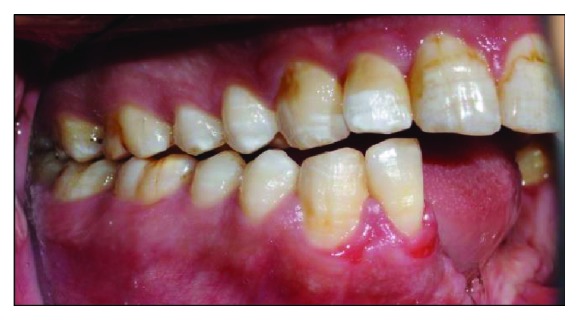
Preoperative right buccal view at maximum intercuspation.

**Figure 6:**
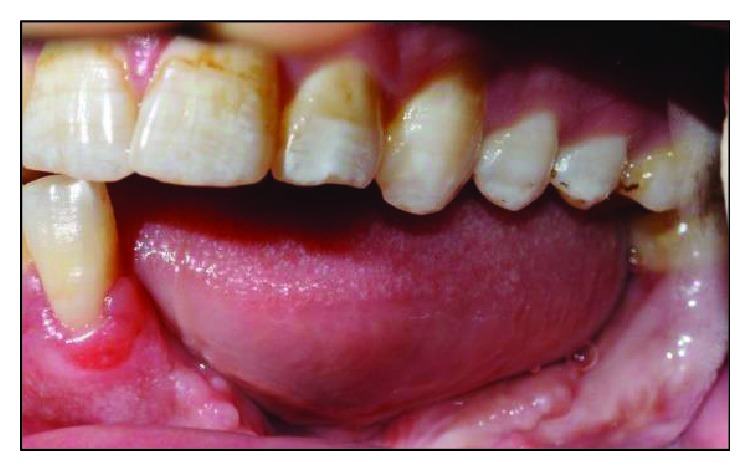
Left buccal view at maximum intercuspation. Note the tongue occupying the edentulous space.

**Figure 7:**
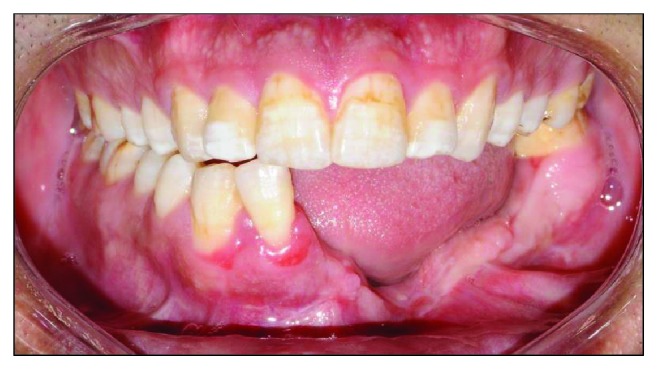
Preoperative frontal view with teeth at maximum intercuspation.

Intraorally, the oral hygiene was fair with the presence of mild gingivitis. The dentition on the maxillary arch was unrestored (Figure 3). The left edentulous mandible was irregular basal bone with firm mucosa covering the bone from 41 until 37. There were marked loss of bony structure horizontally and vertically. This has caused the tongue to occupy the space that used to be occupied by teeth and alveolus in the left mandibular segment (Figures 4, 5, and 6). The healing of the operation site was uneventful (Figures 6 and 7).

**Figure 8:**
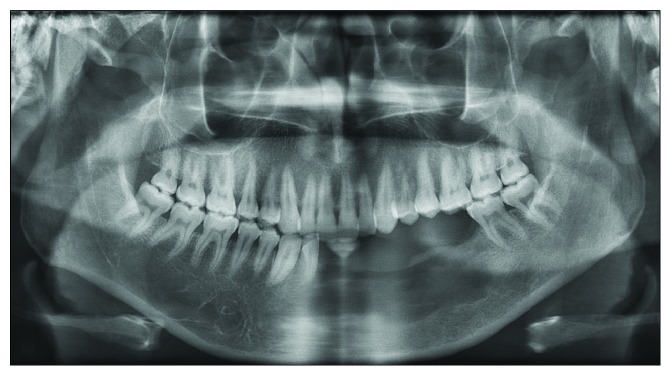
Orthopantomogram.

An orthopantomogram was taken to evaluate the remaining bony structure of the mandible (Figure 8). Radiographically, the operation site (lower left posterior segment) has no abnormalities. The remaining basal bone was adequate in thickness to support the mandible with an irregular margin. There are no radiopaque abnormalities suggestive of new pathology.

### 1.2. Procedures

**Figure 9:**
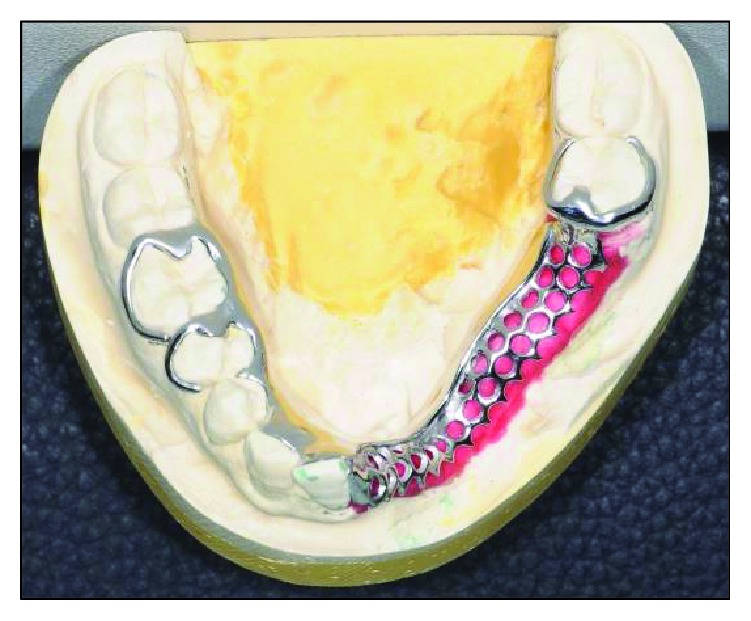
Cobalt-chrome frame constructed on the working model.

Primary impression was made using alginate (Kromopan, Lascod, Illinois, USA) to obtain a set of study cast. A special tray was constructed using a light-cured acrylic resin tray material (plaque photo; W + P Dental, Hamburg, Germany). After border moulding, a secondary impression was taken with a polyether impression (Impregum Penta Soft, 3M-ESPE, Minnesota, USA) and poured with type IV die stone (Vel-Mix, SDS, Kerr, Orange CA) for the construction of the lower cobalt-chrome partial denture frame (Figure 9).

**Figure 10:**
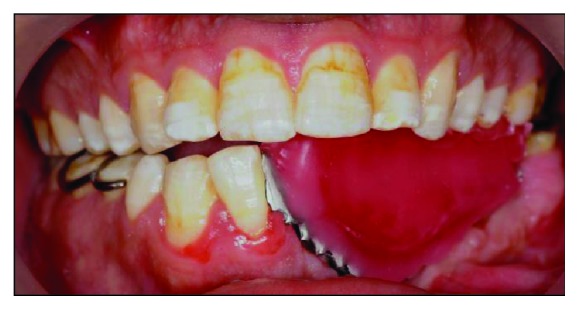
Wax bite record.

**Figure 11:**
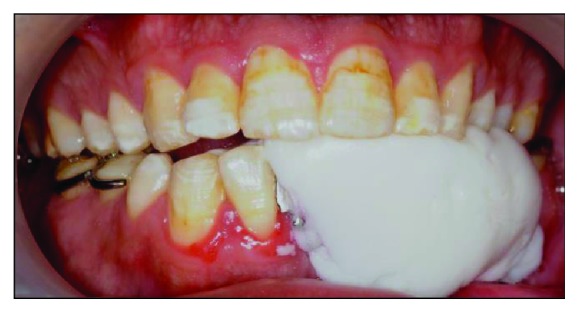
Functional impression obtained to record the neutral zone.

**Figure 12:**
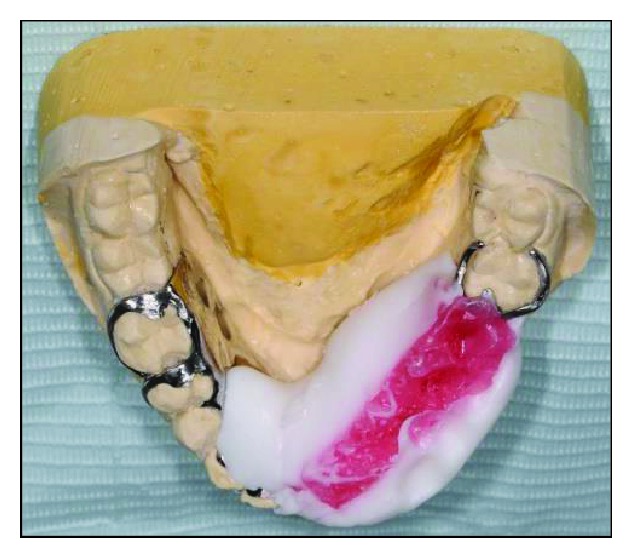
The obtained functional impression.

**Figure 13:**
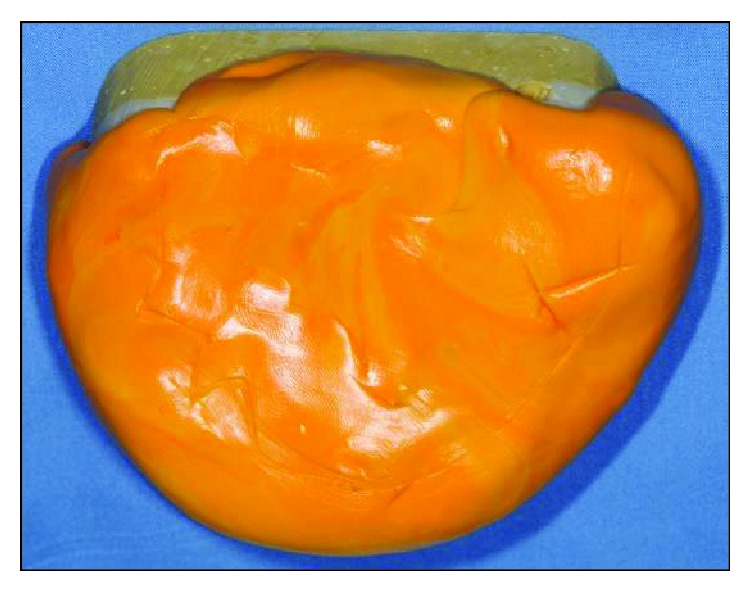
Putty index to serve as a guide for the arrangement of acrylic teeth.

**Figure 14:**
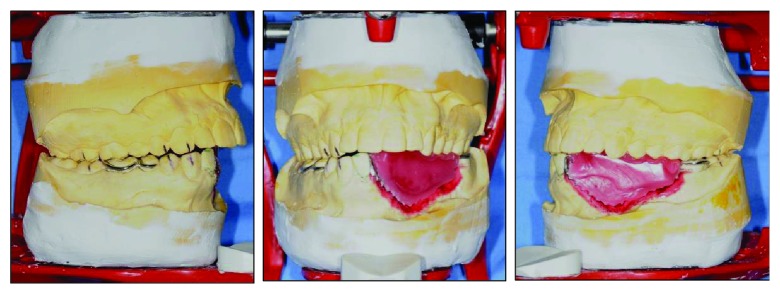
Working model with bite registration mounted on an articulator.

**Figure 15:**
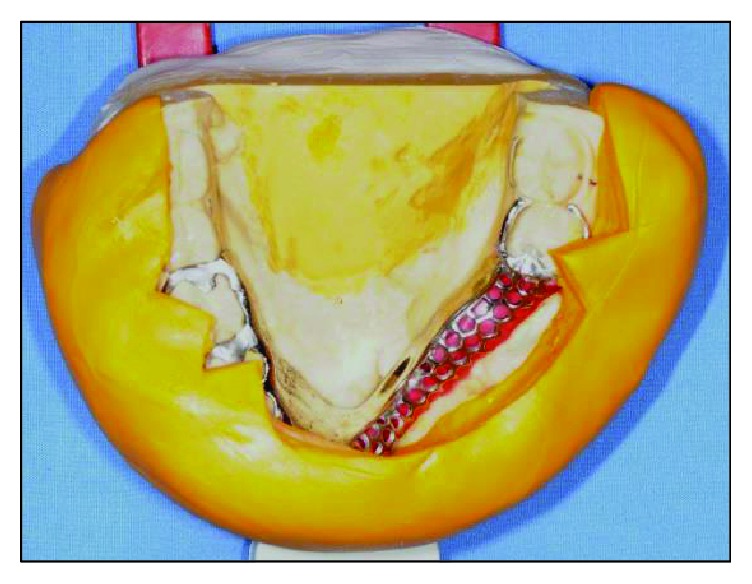
The sectioned putty index showing space for teeth arrangement within the neutral zone.

**Figure 16:**
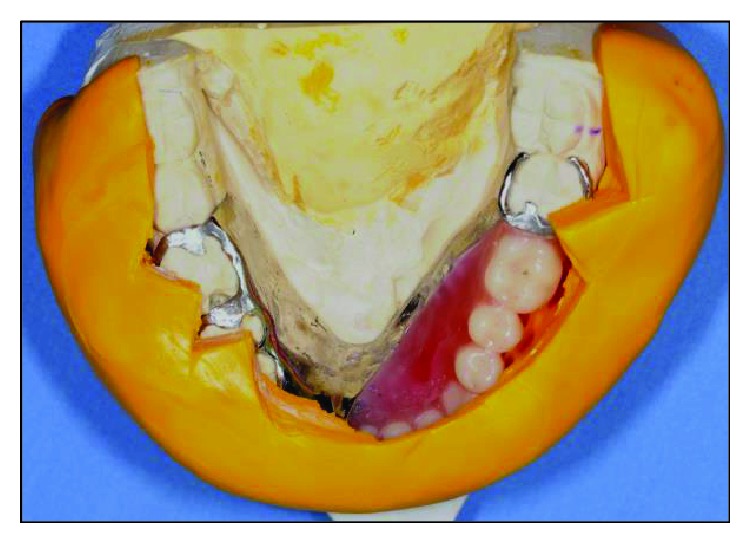
Teeth arranged in the neutral zone.

**Figure 17:**
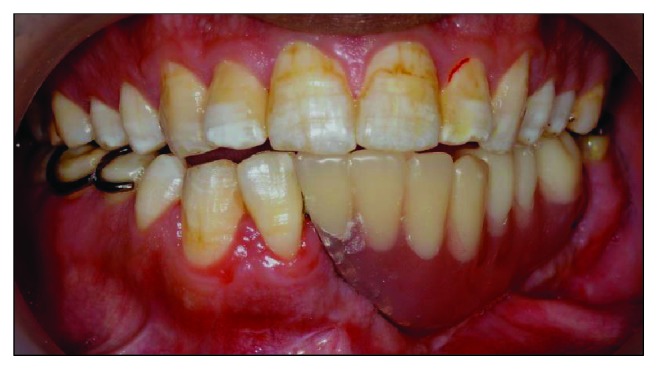
Intraoral facial view at ICP.

**Figure 18:**
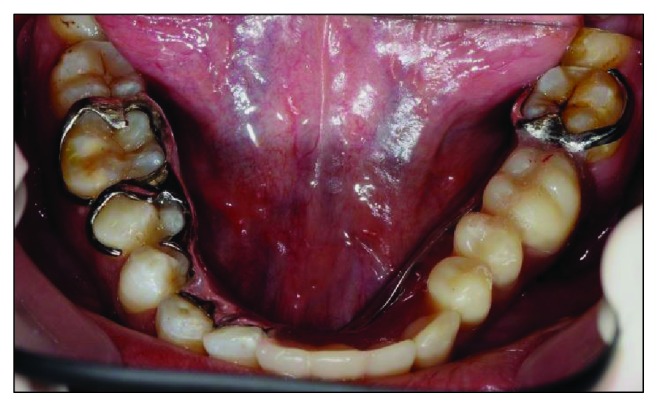
Occlusal view of prosthesis intraorally.

**Figure 19:**
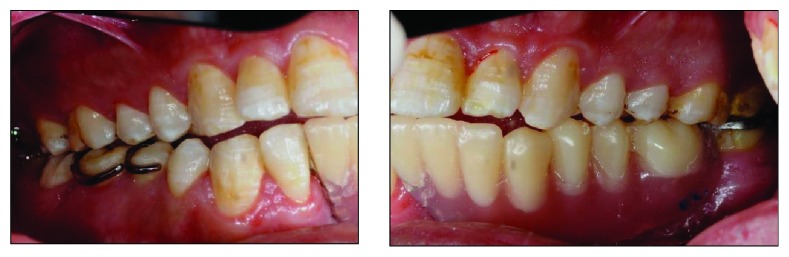
Buccal views after prosthesis delivery.

After the framework try-in procedure, the bite was registered with a block of wax (Figure 10). The borders of the wax rim were adjusted so that the lingual flange was harmonized with the resting and active positions of the floor of the mouth. The buccal width extension was also modified to be a little bit short of the reflection of the cheek. Then, a functional impression procedure was made using a tissue conditioner (F.I.T.T., SDS, Kerr, Orange, CA) to record the neutral zone while the patient was instructed to do basic functional movements in the mouth (Figures 11 and 12). Laboratory putty (Zetaflow Putty, Zhermack SpA, Italy) was then used to record the space obtained from the functional impression (Figure 13). The cast was then mounted (Figure 14) and the putty index was sectioned to visualize the space available for teeth arrangement (Figures 15 and 16). The prosthesis was issued one week after the try-in procedure (Figures 17, 18, and 19).

## 2. Discussion

The keratocystic odontogenic tumour (KCOT), once known as odontogenic keratocyst (OKC), is a benign unicystic or multicystic odontogenic tumour with an aggressive and infiltrative behavior. It is considered a neoplastic lesion due to its locally destructive behavior, with the basal layer of the KCOT budding through connective tissue [4] and the genetics factor involving the tumour suppressor gene PTCH (“patched”) being suppressed [5]. KCOT has also been known to have a high recurrence rate. According to Voorsmit et al. [6] and Irvine and Bowerman [7], the recurrence rate was between 2.5% and 62%. The recurrence rate of KCOT was low when aggressive treatment was done [8]. This happens when the epithelial lining was not completely removed, and this may give rise to new lesion formation. Daughter cells, microcysts, or an epithelial island which may exist in the original walls of the KCOT can contribute to the recurrent lesion [6].

Treatment of KCOT can be divided into conservative and aggressive treatment. Conservative treatment involves enucleation with curettage of marsupialization, which is usually a treatment reserved for a cyst-oriented lesion. Aggressive treatment is usually done using the ostectomy technique and chemical curettage with Carnoy's solution of en bloc resection [9]. The latter technique will result in significant loss of alveolar bone that affects a patient's function as well as aesthetics. In this patient, the alveolar bone loss was vertical as well as horizontal which has left the edentulous site less than ideal for any prosthetic to be emplaced or restorative treatment to be done (Figures 6 and 7).

The patient's left mandibular alveolar bone was completely removed and the basal bone was preserved (Figures 6 and 8). This allows the restorative dentist to have a foundation to start rehabilitation. The basal bone can act as a guide for bone augmentation, and it can also preserve the patient's facial profile. This can minimize the psychological effect due to facial disfigurement after surgical removal of the lesion and reconstruction. If the left body of the mandible was completely removed, the surgeon needs to reconstruct the defect by an autogenous bone graft from a distant site [10]. Nowadays, a surgeon prefers the use of a vascularized bone graft in mandibular reconstruction since it promises a success rate of up to 90% [11]. The patient was not receiving any bone augmentation since the basal bone was intact.

**Figure 20:**
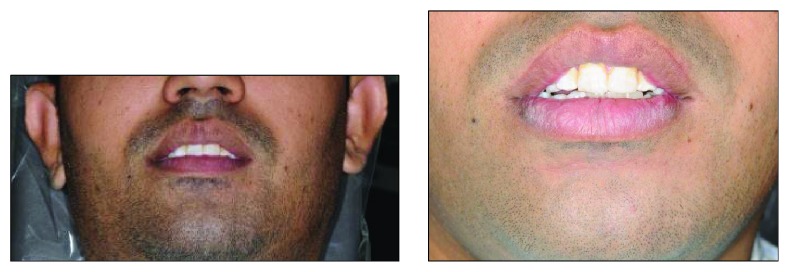
Postoperative photograph. Note that the collapse of the buccal cheek has been restored symmetrically.

Several options were given to the patient regarding the rehabilitation of his left mandibular defect. An implant that retained fixed partial dentures is a promising option given its high success rate. Prior to that, the left body of the mandible needs to be augmented to restore the bone height and width to provide a sound platform for placement. This is to ensure that the implant is not subjected to a high-tipping force resulting from an increased coronal : root ratio in implant restoration. Since the recurrence rate of KCOT was high, it is wise to defer the implant treatment and observe for any sign of recurrence. The recurrence of KCOT can take place up to 5–7 years after treatment [9] and prolonged follow-up is crucial. The patient wished to have a long-term provisional replacement for his missing teeth on the left of the mandible and a cobalt-chrome partial denture was suggested. The cobalt-chrome partial denture was chosen due to the rigidity of the framework and superior surface polishability compared to an acrylic partial denture. The partial denture can also serve as a template for bone augmentation and for the placement of future implants. The partial denture can restore the function of the patient and will improve his aesthetics and confidence in dealing with society (Figure 20).

In this patient, a partial denture needs to be constructed in a neutral zone, a space that is free of muscle movement. This space is a result of a balanced interaction between the tongue's outward movement together with buccal mucosa and lip inward movement during this function [12]. By placing the denture teeth and flange in the neutral zone, this can avoid breaching the buccal space occupied by the cheek and lip as well as the lingual space occupied by the tongue, creating a partial denture that exists in muscle balance. Although this practice can usually be done on a full lower denture, in this case since the alveolar bone has a marked loss in the vertical and horizontal direction together with a collapsed left lip and reduced cheek support, the neutral-zone impression serves as a guide for teeth arrangement.

Upon issue of the lower partial denture, the patient was very satisfied with the result. He was happy to have his lower lip and cheek restored to the original contour and was satisfied with his smile (Figure 20). He was able to eat comfortably with the partial denture. His speech was improved after the prosthesis was issued. The patient was given instructions to maintain good oral hygiene and to observe the left edentulous site for any sign of recurrence or to report any sign and symptom immediately for further management. He was given a follow-up review every six months.

## 3. Conclusions

In the present case, we demonstrate the construction of a removable partial denture recorded at a neutral zone resulting in an adequate restoration of a patient's aesthetics and function with minimal displacement from the normal soft tissue movement. The neutral-zone impression technique also allows a dental technician to arrange the acrylic teeth in an area with minimal deviation. The marked loss of alveolar bone secondary to surgical removal can give difficulty to the technician to arrange acrylic teeth in the actual position, which can later cause discomfort to the patient and may result in partial denture instability.
